# QTL mapping of Fusarium head blight resistance in three related durum wheat populations

**DOI:** 10.1007/s00122-016-2785-0

**Published:** 2016-09-23

**Authors:** Noemie Prat, Camille Guilbert, Ursa Prah, Elisabeth Wachter, Barbara Steiner, Thierry Langin, Olivier Robert, Hermann Buerstmayr

**Affiliations:** 1 Department of Agrobiotechnology, Institute of Biotechnology in Plant Production, BOKU-University of Natural Resources and Life Sciences Vienna, Konrad Lorenz Str. 20, 3430 Tulln, Austria; 2 INRA, UBP, Genetics, Diversity and Ecophysiology of Cereals, GDEC, 63039 Clermont-Ferrand Cedex 2, France; 3 Florimond-Desprez, 3 rue Florimond-Desprez, BP 41, 59242 Cappelle-en-Pevele, France

## Abstract

*****Key message***:**

**The QTL** ***Fhb1*** **was successfully introgressed and validated in three durum wheat populations. The novel germplasm and the QTL detected will support improvement of Fusarium resistance in durum wheat.**

**Abstract:**

Durum wheat (*Triticum durum* Desf.) is particularly susceptible to Fusarium head blight (FHB) and breeding for resistance is hampered by limited genetic variation within this species. To date, resistant sources are mainly available in a few wild relative tetraploid wheat accessions. In this study, the effect of the well-known hexaploid wheat (*Triticum aestivum* L.) quantitative trait locus (QTL) *Fhb1* was assessed for the first time in durum wheat. Three F7-RIL mapping populations of about 100 lines were developed from crosses between the durum wheat experimental line DBC-480, which carries an *Fhb1* introgression from Sumai-3, and the European *T. durum* cultivars Karur, Durobonus and SZD1029K. The RILs were evaluated in field experiments for FHB resistance in three seasons using spray inoculation and genotyped with SSR as well as genotyping-by-sequencing markers. QTL associated with FHB resistance were identified on chromosome arms 2BL, 3BS, 4AL, 4BS, 5AL and 6AS at which the resistant parent DBC-480 contributed the positive alleles. The QTL on 3BS was detected in all three populations centered at the *Fhb1* interval. The *Rht*-*B1* locus governing plant height was found to have a strong effect in modulating FHB severity in all populations. The negative effect of the semi-dwarf allele *Rht*-*B1b* on FHB resistance was compensated by combining with *Fhb1* and additional resistance QTL. The successful deployment of *Fhb1* in *T. durum* was further substantiated by assessing type 2 resistance in one population. The efficient introgression of *Fhb1* represents a significant step forward for enhancing FHB resistance in durum wheat.

**Electronic supplementary material:**

The online version of this article (doi:10.1007/s00122-016-2785-0) contains supplementary material, which is available to authorized users.

## Introduction

Fusarium head blight (FHB), caused mainly by *Fusarium graminearum* and *Fusarium culmorum*, is one of the major fungal diseases affecting wheat production almost worldwide (Parry et al. 1995). The direct consequences of FHB are yield losses and seed quality reductions in both common wheat (*Triticum aestivum*) and durum wheat (*Triticum durum*) (McMullen et al. 2012). The contamination of infected grains with Fusarium mycotoxins is highly problematic, rendering harvests unfit for food and feed (Pestka 2010; Covarelli et al. 2014). Mycotoxin contamination is of particular concern in durum wheat as it is mainly utilized for human consumption. Host plant resistance is considered pivotal for an integrated plant protection strategy to control and reduce FHB damages (Gilbert and Haber 2013).

FHB is a complex disease and its response shows polygenic inheritance modulated by environmental factors with significant genotype-by-environment interactions (Buerstmayr et al. 2009; Löffler et al. 2009). Several components of resistance have been defined (Schroeder and Christensen 1963; Mesterházy 1995), among which resistance to initial infection (type 1) and resistance to fungal spread within infected spikes (type 2) are commonly accepted and have been widely investigated in QTL mapping studies. Under field conditions, the overall FHB resistance is usually assessed through scoring of disease severity after spray inoculation and is considered to reflect the genotypic response during natural epidemics. Both active and passive mechanisms influence FHB resistance (Mesterházy 1995). The latter include morphological and developmental features which affect primary fungal infection and/or disease development through disease escape mechanisms. Plant height is one of the foremost morphological traits affecting FHB response and the widely deployed Norin 10 semi-dwarfing *Rht* alleles, namely *Rht*-*B1b* and *Rht*-*D1b*, have been found associated with increased FHB severity under field conditions in common wheat (Hilton et al. 1999; Miedaner and Voss 2008; Voss et al. 2008) and in durum wheat (Buerstmayr et al. 2012). Their effect on FHB development may be imputed to plant height per se and differences in canopy structure (Yan et al. 2011) as well as to pleiotropic physiological effects of the *Rht* genes and/or the presence of tightly linked genes (Srinivasachary et al. 2009; Saville et al. 2012).

Compared to common wheat, limited efforts have been dedicated to improve FHB resistance in durum wheat (Buerstmayr et al. 2009). Most current durum wheat cultivars are highly susceptible and breeding progress is hampered by the narrow genetic variation for FHB resistance in durum wheat elite germplasm. Extensive screening of large germplasm collections identified only few durum landraces with improved levels of resistance (Elias et al. 2005; Talas et al. 2011; Huhn et al. 2012). Alternative sources of resistance have been screened in the related tetraploid species of *Triticum turgidum* to identify resistance donors for breeding (Buerstmayr et al. 2003b; Oliver et al. 2007, 2008). A relatively small number of QTL mapping studies aimed at dissecting the genetic architecture of FHB resistance in tetraploid wheat to date and have been recently reviewed by Prat et al. (2014). QTL descending from *Triticum dicoccoides* accessions Israel A, PI478742, Mt. Hermon#22, and Mt. Gerizim#36 have been identified on chromosomes 3A (Otto et al. 2002; Gladysz et al. 2007; Chen et al. 2007; Buerstmayr et al. 2013; Zhu et al. 2016b), 4A (Gladysz et al. 2007), 6B (Buerstmayr et al. 2013) and 7A (Kumar et al. 2007). *Triticum dicoccum* accessions PI 41025, Td-161 and BGRC3487 provided resistance QTL on 3A, 3B, 4B, 5A, 6A, 6B, 7A and 7B (Buerstmayr et al. 2012; Ruan et al. 2012; Zhang et al. 2014). *Triticum carthlicum* accession Blackbird contributed one resistance QTL mapping to 6B (Somers et al. 2006). The dissection of the genetic architecture of resistant *T. durum* Tunisian landraces by association mapping located a QTL on 3B (Ghavami et al. 2011). In mapping studies based on crosses between susceptible durum cultivars and other resistant sources, a few resistance-conferring QTL alleles were contributed by the susceptible durum parents, notably those on chromosomes 2A, 2B, 3B and 5B from cultivars Ben (Zhang et al. 2014), Strongfield (Somers et al. 2006), Floradur (Buerstmayr et al. 2012) and Lebsock (Ghavami et al. 2011), respectively.

The QTL detected in tetraploid wheat have failed so far to provide similarly high levels of resistance such as *Fhb1*, the major resistance QTL identified in common wheat cultivar Sumai-3 (Waldron et al. 1999; Anderson et al. 2001). Near diagnostic markers for *Fhb1* are available (Liu et al. 2008; Schweiger et al. 2016) and have been successfully implemented into applied wheat breeding using marker-assisted selection (Anderson et al. 2007; Wilde et al. 2007; Salameh et al. 2011). Notwithstanding, the consequence of transferring this major QTL into tetraploid wheat has not been communicated until now. Here, we report on the effect of *Fhb1* in three biparental populations that have been generated by crossing line DBC-480, an FHB-resistant experimental durum line possessing the *Fhb1* allele from Sumai-3, with a modern European durum breeding line and two current durum cultivars. We also show the association of plant height with FHB resistance, and more specifically the effect of *Rht*-*B1* and its interaction with *Fhb1* on disease severity in durum wheat.

## Materials and methods

### Plant materials

Three mapping populations comprising 111, 100 and 100 F_7_ RILs were developed by single seed descent from crosses of the tetraploid resistant line DBC-480 to the susceptible European *T. durum* cultivars Karur (KD) and Durobonus (DD) and the advanced breeding line SZD1029K (SD), respectively. Karur and Durobonus are registered varieties bred by RAGT, France (registered 2002), and Saatzucht-Donau, Austria (registered 2004), respectively. The breeding line SZD1029K was provided by Saatzucht-Donau for this study. The experimental line DBC-480 was developed at IFA-Tulln, Austria, by four generations of marker-assisted backcrossing of the highly resistant *T. aestivum* cultivar Sumai-3 into the background of the Austrian *T. durum* variety Semperdur and subjected to rigorous phenotypic selection for improved FHB resistance in field trials (details not shown). The presence of the resistant allele at *Fhb1* was verified using the SSR markers *Xgwm389*, *Xgwm533* and *Xgwm493*. Karur, Durobonus and SZD1029K possess the semi-dwarfing allele *Rht*-*B1b*, while DBC-480 is a tall line that harbors the *Rht*-*B1a* wild-type allele.

### FHB resistance phenotyping

The three mapping populations along with their parental lines were evaluated in multiple field experiments at IFA-Tulln, Austria (16°04,16′E, 48°19,08′N, 177 m above sea level) in 2013, 2014 and 2015. Experiments were laid out as randomized complete block designs with two blocks in 2013 and three blocks in 2014 and 2015. Plots consisted of single rows in 2013 and double rows of 1 m length at 17 cm spacing in 2014 and 2015. Sowing of the individual blocks was performed in early spring and staggered 1–2 weeks apart leading to slightly different flowering dates between the blocks. Management of the field trials was conducted following good agronomical practice as described in Buerstmayr et al. (2002). At anthesis, trials were spray inoculated using a motor-driven backpack sprayer in the late afternoons with the virulent DON-producing *F. culmorum* isolate Fc91015 at a conidial concentration of 2.5 × 10^4^ ml^−1^. Inoculum suspension was prepared by utilizing the protocol described in Buerstmayr et al. (2000). Aliquots of conidia stock solutions were stored at −30 °C then thawed at 37 °C and diluted with deionized water to achieve the desired final spore concentration just prior to inoculation. Inoculations were performed within each block on all plots, starting when 50% of the plants in the earliest plot of a block reached anthesis. Inoculations were repeated at 2-day intervals and ended 2 days after the last plot of the block flowered, resulting in up to six inoculum applications per block. At each inoculation cycle, about 100 ml m^−2^ of conidial suspension was sprayed onto the durum wheat heads. The crop canopy was kept moist by mist irrigating during 20 h after inoculations to facilitate spore germination and infection. FHB severity was visually estimated as the percentage of infected spikelets within each plot on days 14, 18, 22 and 26 after anthesis. In 2013, scoring was performed at two time points: 18 and 26 days after anthesis. The area under the disease progress curve (AUDPC) was calculated and used as an integrated measure of overall disease severity as described by Buerstmayr et al. (2000). At all experimental plots, plant height (PH) was measured in centimeter and flowering date was recorded and converted into number of days after May 1.

The KD population and the respective parental lines Karur and DBC-480 were also tested in the greenhouse for FHB spread within the spike (type 2 resistance) using single-floret inoculation in three unreplicated greenhouse trials at Florimond-Desprez (France) in winter 2015 (GH1) and at IFA-Tulln in summer 2016 (GH2 and GH3). Seeds were germinated in multi-trays and subjected to a cold treatment at 5 °C for 1 week. Ten seedlings per line were planted in 7.5-l pots (23.5 cm diameter, 23 cm height) filled with a standard potting mix consisting of 70% recycled compost, 28% peat and 2% silica sand. Pots were designated as experimental units and arranged in a randomized design. The temperature in the greenhouse was maintained at 22/18 °C (day/night) with a 16-h photoperiod. Management of the greenhouse trial was essentially as described by Buerstmayr et al. (2013). Inoculations were performed at anthesis by pipetting 10 µl of conidia suspension between the lemma and palea of the four outer florets of two central spikelets per spike using the same inoculum preparation and concentration as for the field experiments. High humidity was ensured to promote fungal infection by covering the spikes with translucent polyethylene bags for 24 h. Type 2 resistance was assessed as the percentage of infected spikelets per spike (PIS) measured at 24 days post-inoculation by counting the number of infected spikelets and the total number of spikelets per spike. On average, eight spikes per genotype were inoculated in each experiment. Plant height was recorded at each greenhouse pot.

### Phenotypic data analysis

Statistical analysis was performed in R 3.1.3 (R Core Team 2015) using the lme4 package for mixed model analysis (Bates et al. 2015). For each trait under investigation, a linear mixed model was fitted for each population with all three experiments combined: (1) $P_{ijk} \; = \mu \; + \;G_{i} \; + \;E_{j} \; + \;E_{j} (R_{k} )\; + \;G_{i} \; \times \;E_{i} \; + \;e_{ijk} ,$ where $P_{ijk}$ is the phenotypic value, µ the population mean, $G_{i}$ the effect of the ith genotype, $E_{j}$ the effect of the jth experiment, $E_{j} (R_{k} )$ the effect of the kth replicate within the jth experiment, ${\text{G}}_{\text{i}} \times {\text{E}}_{\text{i}}$ the ijth effect of the genotype-by-experiment interaction and $e_{ijk}$ designated the residual. The genotype effect was treated as fixed and all other terms as random effects. For single experiments, a reduced linear mixed model was fitted: (2) $P_{ik} \; = \;\mu \; + \;G_{i} \; + \;R_{k} \; + \;e_{ik}$, where $P_{ik}$ is the phenotypic value, µ the population mean, $G_{i}$ the effect of the ith genotype, $R_{k}$ the effect of the kth replicate (block) and $e_{ik}$ the residual. The genotype effect was again treated as fixed and the replication as random effect.

Fixed and random effects of the models were tested one by one using the likelihood ratio test (LRT). Best linear unbiased estimates (BLUES) of each genotype were computed for the different phenotyped traits according to model (1) for the analysis across experiments and according to model (2) for an analysis within individual experiments. BLUES calculated across experiments are also referred to as overall means. Broad-sense heritability ($H^{2}$) was estimated using variance components determined by the restricted maximum likelihood (REML) method setting all effects as random and based on the equation $H^{2} = \sigma_{G}^{2} /(\sigma_{G}^{2} \; + \;\sigma_{G \times E}^{2} /m\; + \;\sigma_{e}^{2} /p),$ where $\sigma_{G}^{2}$ denotes the genotypic variance, $\sigma_{{{\text{G}} \times {\text{E}}}}^{2}$ the genotype-by-experiment interaction variance, $\sigma_{e}^{2}$ the error variance, m the number of experiments and p the total number of replications across experiments (Holland et al. 2003).

### Marker data and genetic map construction

Genomic DNA was extracted from fresh leaves of ten pooled plants of each line using a simplified CTAB-based procedure modified from Saghai-Maroof et al. (1984). High-density genotyping of all individuals was performed using genotyping-by-sequencing (GBS) with the DArTseq platform (DArT PL, Canberra, Australia). Markers identified by the DArTseq assay include SNPs as well as the presence–absence variations (PAV) (Li et al. 2015). Markers were filtered based on a reproducibility ≥95%. Furthermore, PAV with ≥10% missing data and SNP markers with ≥10% missing data or heterozygotes were removed for each population separately. Markers showing significant (*p* < 0.01) segregation distortion were also discarded. Finally, a total of 7965, 4150 and 6235 high-quality polymorphic DArTseq markers were available for mapping in the KD, DD and SD populations, respectively. All lines were genotyped with the two selected markers *Xbarc147* (Song et al. 2005) and *Xumn10* (Liu et al. 2008) that are known to be linked with *Fhb1* as well as with allele-specific markers for *Rht*-*B1* (Ellis et al. 2002).

### Linkage map construction

Linkage maps for each population were constructed using the MSTmap algorithm (Wu et al. 2008) included in the R package ASMap v0.4 (Taylor and Butler 2015). The objective function was set to minimize the sum of recombination events between markers for map construction. In a first step, robust linkage groups where constructed using a *p* value threshold set to 1 × 10^−8^, and the assignment of the linkage groups to chromosome was performed by comparing the location of markers to the wheat DArTseq consensus map provided by DArT PL (A. Kilian, Diversity Arrays Technologies, personal communication, 2016). In a second step, genotypic data were pooled on a chromosome basis and regrouped at a less stringent threshold using a *p* value of 1 × 10^−6^. Distances were calculated with the Kosambi mapping function. Genetic maps were drawn on MapChart software (Voorrips 2002) and collinearity among the individual maps was checked.

### QTL mapping

Quantitative trait loci analysis was performed for each trait with the BLUES calculated for each individual experiment and across experiments using the R package R/qtl (Broman et al. 2003). Missing genotypic information was imputed using the multiple imputation method of Sen and Churchill (2001). The main effect QTL were detected by performing interval mapping and composite interval mapping via Haley–Knott regression. For composite interval mapping, the number of marker covariates was selected by a forward approach, while setting a window size of 10 cM. LOD significance threshold for type I error rate *α* = 0.05 were obtained for each trait and experiment based on a 1000 permutations test. Significant QTL were subsequently fitted using a multiple QTL model. The existence of further QTL and the presence of QTL-by-QTL interaction were tested using the *addqtl* and *addint* functions, respectively. The final multiple QTL model was fitted against the null model by ANOVA and the percentage of phenotypic variance explained by each QTL, the additive effects as well as LOD scores were estimated. Confidence intervals were defined for each QTL by calculating a 1.5-LOD support interval.

## Results

### Trait variations and correlations

**Fig. 1:**
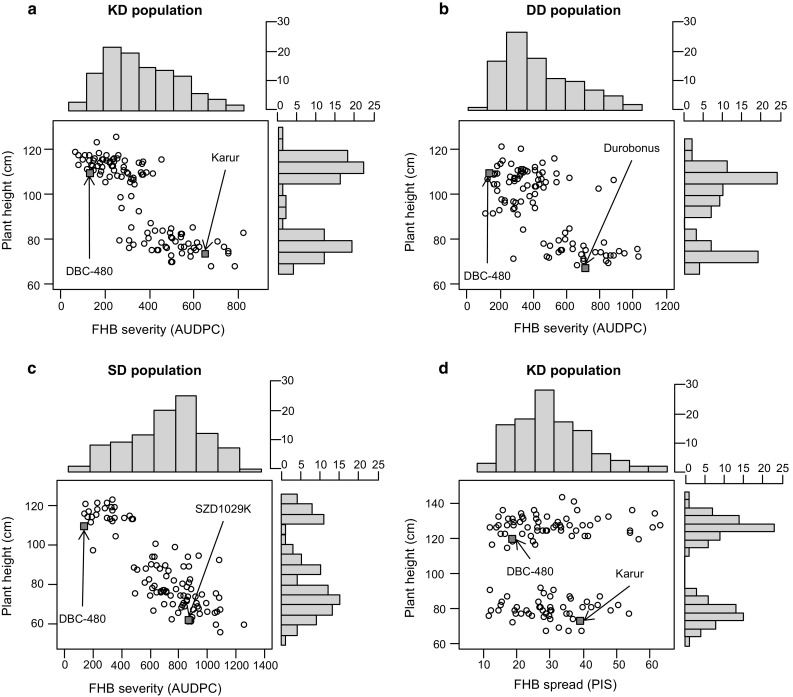
Scatter plots and marginal histograms of frequency distribution of overall means for FHB severity (AUDPC) against plant height (cm) measured in the field trials for each population (**a**–**c**) and for FHB spread (PIS) again plant height (cm) measured in the greenhouse trials for the KD population (**d**). Parents are indicated by *arrows*

Evaluation of FHB severity was performed on three biparental populations in artificially inoculated field trials to investigate the relevant factors that play a role in reducing FHB disease under natural conditions. In all experiments, the common resistant parent DBC-480 was significantly less diseased than the susceptible parents. The average AUDPC value across experiments of the resistant parental line DBC-480 was 125 (corresponding to an average of 12% symptomatic spikelets 26 days after flowering), while Karur, Durobonus and SZD1029K had approximately fivefold higher AUDPC values (Table 1). Large variation was observed within each population (Fig. 1), but also between populations where the average FHB severity was lowest in the KD population and highest in the SD population. Transgressive segregation was observed in all populations and some lines showed lower disease symptoms than the resistant parent DBC-480, although these differences were not statistically significant. Disease pressure between experiments was comparable in 2013 and 2014, while the experiment of 2015 showed overall higher symptoms. AUDPC broad-sense heritability for means across experiments was high and within the same range for the three populations (0.74 < *H* ^2^ < 0.89), as in all cases, genotypic variances were higher than variances due to genotype × experiment interaction and residual error (Online Resource 1). Significant genotypic effects for all traits were revealed by ANOVA.

**Table 1:** Means of parents and mean, minimum and maximum values of populations, least significant differences at *α* < 0.05 (LSD_0.05_) and broad-sense heritability coefficient (*H* ^2^) or repeatability of analyzed traits in field and greenhouse (GH) experiments

	Parents				Population				
	DBC-480	Karur	Durobonus	SZD1029K	KD				
					Mean	Min	Max	LSD_0.05_	*H* ^2^
FHB severity (AUDPC)									
Overall mean	125	642	693	845	360	65	816	117	0.89
2013	126	668	601	874	272	23	830	258	0.66^c^
2014	54	457	567	519	289	16	781	175	0.87^c^
2015	200	805	917	1130	516	94	1128	185	0.92^c^
FHB spread (PIS^b^)	18.3	38.4	–	–	30.2	11.6	62.6	25.4	0.51
Flowering date^a^	40.3	40.1	40.1	40.1	39.6	37.6	41.9	1.1	0.67
Plant height (cm) in field	110	73	67	61	97	67	126	5	0.98
Plant height (cm) in GH	119	71	–	–	105	65	143	12	0.97

	Population									
	DD					SD				
	Mean	Min	Max	LSD_0.05_	*H* ^2^	Mean	Min	Max	LSD_0.05_	*H* ^2^
FHB severity (AUDPC)										
Overall mean	451	110	1152	142	0.78	667	131	1237	136	0.74
2013	292	47	699	210	0.61^c^	401	47	915	249	0.69^c^
2014	240	32	768	151	0.84^c^	328	28	903	165	0.86^c^
2015	791	201	1718	302	0.93^c^	1257	251	2031	264	0.96^c^
FHB spread (PIS^b^)	–	–	–	–	–	–	–	–	–	–
Flowering date^a^	39.3	36.5	41.7	1.2	0.76	41	38.3	43.3	1.1	0.81
Plant height (cm) in field	95	68	121	6	0.97	85	54	123	5	0.99
Plant height (cm) in GH	–	–	–	–	–		–	–	–	–

^a^Number of days from May 1 to anthesis

^b^Percentage of infected spikelets

^c^Repeatability

To evaluate specific type 2 component of resistance as conferred by *Fhb1*, the percentage of infected spikelets per spike (PIS) was measured in three glasshouse experiments for the KD population. Heritability for PIS was moderate, *H* ^2^ = 0.51, although ANOVA showed significant genotype effects for the 111 RILs (Table 1, Online Resource 1). Some of the RILs showed transgressive segregation for type 2 resistance, although only significant for higher susceptibility. As expected, DBC-480 showed less disease symptoms than Karur with an average of 18.3 and 38.4% PIS, respectively (Fig. 1). Weak but significant correlation was observed between the means of FHB spread and FHB severity (*r* = 0.2, *p* < 0.05).

Variation for plant height was apparent in the three populations (Fig. 1). The susceptible parents Karur, Durobonus and SZD1029K were 38–49 cm shorter than the resistant donor DBC-480. PH showed a bimodal frequency distribution in the KD and DD populations, while a trimodal distribution was displayed in the SD population (Fig. 1). FHB severity was negatively correlated with PH within all populations (Table 2), accordingly shorter plants showed higher FHB severity. On the contrary, FHB spread after point inoculation (PIS) showed no significant correlation with PH. Distribution of date of anthesis showed continuous variation in all populations, although no significant difference in flowering date was observable among the parents. FHB severity and flowering date were significantly positively correlated in the SD population across experiments, while a weak negative correlation and no evidence for significant correlation were observed in the KD and DD populations, respectively (Table 2). Within individual experiments, however, correlation analysis showed no significant association between disease severity and date of anthesis in 2013 and 2015 for the KD population in 2013 and 2014 for the DD population and in 2014 for the SD population (Online Resource 2).

**Table 2:** Pearson correlation coefficients between FHB severity (AUDPC), plant height (cm) and flowering date (days after May 1) for the overall means

	FHB severity (AUDPC)		
	KD	DD	SD
Plant height	−0.82***	−0.67***	−0.85***
Flowering date	−0.20*	0.15 n.s.	0.43***

n.s. non-significant

* *p* < 0.05

*** *p* < 0.001

### QTL analysis

#### Generation of linkage maps

7975, 4153 and 6242 polymorphic markers were generated from DARTseq and SSR marker data for the KD, DD and SD populations, respectively. Of these markers, 1064 were common across all three populations. The number of markers within maps for the KD, DD and SD populations was reduced to 1609, 1052 and 1006 unique loci, respectively. The total map lengths were 2806, 1781 and 2219 cM with an average marker distance of 1.9, 1.7 and 2.5 cM for the KD, DD and SD populations, respectively. Each linkage group could be unambiguously assigned to a chromosome based on the wheat DArTseq consensus map. Alignment to the consensus map showed low-coverage regions for the DD and SD populations on chromosomes 1A and 3A and on chromosomes 5A and 7A for the DD population. Despite that, all chromosomes were represented (Online Resource 3).

#### QTL analysis for FHB severity

**Fig. 2:**
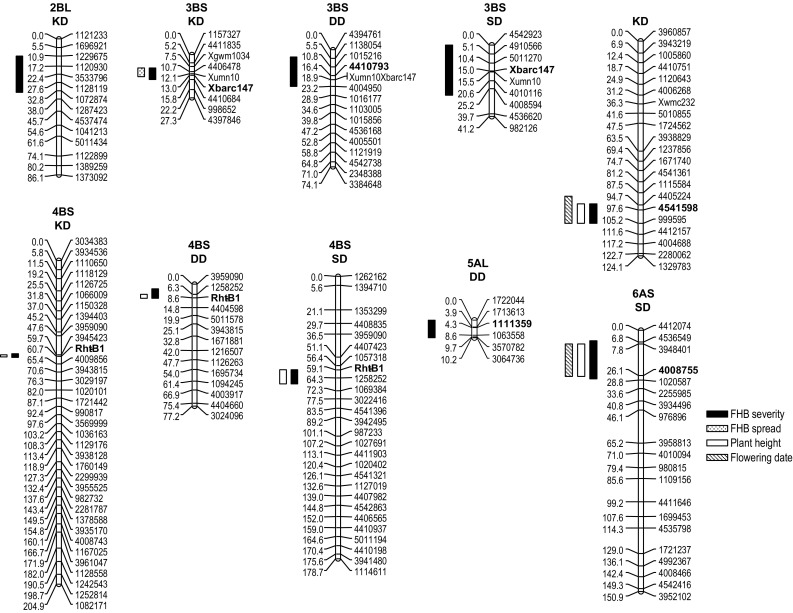
Linkage maps and positions of QTL for FHB severity (AUDPC), FHB spread (percent of infected spikelets PIS) and coinciding morphological/developmental traits of the three populations based on overall means. For readability, only selected markers are shown. Loci closest to the QTL peak of FHB severity are in *bold*. QTL *bars* span an LOD drop of 1.5 from maximum LOD

QTL analysis conducted in individual populations identified a total of six genomic regions associated with FHB severity on chromosome arms 2BL, 3BS, 4AL, 4BS, 5AL and 6AS (Table 3). The resistant parent DBC-480 contributed to the resistance, improving alleles at all loci. Linkage groups and confidence intervals of QTL are shown in Fig. 2. For reading ease, only selected markers at about 5 cM distances are displayed, while more detailed information including all mapped markers with their positions can be found in Online Resource 4. The two genomic regions on 3BS and 4BS were found to be repeatedly associated with FHB resistance at the same location in all three populations. The major QTL on 4BS co-localized with the *Rht*-*B1* locus, which explained 64, 38 and 19% of the total phenotypic variance in the KD, DD and SD populations, respectively. On chromosome 3BS, the QTL mapped to marker positions *Xbarc147* and *Xumn10*, which signposts the position of the introgressed *Fhb1* and was detected for the analysis across experiments in all populations. The 3BS QTL was found consistently in all experiments for the KD population, while it was significant in two out of three experiments for the DD population and in one experiment for the SD population. The effects of the contrasting alleles at the *Fhb1* and *Rht*-*B1* loci, as well as the effect of allelic combinations at these loci for the overall mean FHB severity are illustrated for each population in Online Resource 5. Analysis revealed further QTL specific to individual populations. Two major QTL were detected in the SD population on 4AL and 6AS explaining 19 and 25% of the total phenotypic variation. Both QTL overlapped with QTL associated with plant height and flowering date. In the DD population, a QTL on 5AL was found in the analysis across experiments where it contributed to 6% of the phenotypic variation and had a stronger effect in 2013 explaining 15% of the phenotypic variation while it was not significant in 2014 and 2015. A small effect QTL was detected on 2BL in the KD population which contributed to 4% of the phenotypic variance and was significant in 2014 and for the across-experiments analysis. There was no evidence for epistatic QTL interactions in any of the analyses; QTL for FHB severity acted thus in an additive manner.

**Table 3:** Locations and estimates of QTL for FHB severity (AUDPC) using multiple QTL mapping

Population	Chr.	Closest marker	Overall mean			2013		2014		2015	
			Add^a^	%PV^b^	LOD^c^	%PV^b^	LOD^c^	%PV^b^	LOD	%PV^b^	LOD^c^
KD	2BL	1072874	37	4.3	4.8	–	–	7.2	5.5	–	–
DD	3BS	4410793	86	14.0	8.7	–	–	16.0	4.7	12.2	7.6
KD	3BS	Xbarc147	60	11.1	10.8	14.1	5.5	12.8	9.0	6.1	5.6
SD	3BS	Xbarc147	60	5.0	3.3	–	–	8.1	3.8	–	–
SD	4AL	4541598	123	18.8	10.4	–	–	15.4	6.7	14.4	6.6
DD	4BS	RhtB1	156	38.4	18.6	18.8	6.0	26.0	7.1	55.5	23.1
KD	4BS	RhtB1	140	64.2	35.0	29.1	10.2	47.6	23.7	69.0	33.2
SD	4BS	RhtB1	126	19.4	10.7	–	–	10.3	4.7	16.0	7.2
DD	5AL	1111359	59	6.2	4.3	15.0	5.0	–	–	–	–
SD	6AS	4008755	139	24.9	12.9	28.0	6.9	24.6	9.8	25.4	10.6

^a^Positive additive effects denote trait-increasing effect of the DBC-480 allele; additive effects were estimated as half the difference between phenotype averages for the homozygote

^b^Percentage of phenotypic variance explained by the QTL

^c^LOD (logarithm of the odds) above the threshold at the 0.05 level of probability obtained through a 1000-iteration permutation test

#### QTL analysis for FHB spread

QTL detection for FHB spread in the KD population identified a QTL on 3BS which peaked at the SSR marker *Xbarc147* and was thus located in the same region as FHB severity 3BS QTL, matching likewise with the *Fhb1* locus. The resistance-conferring allele was derived from DBC-480. The QTL was consistently detected in all three individual experiments and for the analysis across all experiments in which it explained 33% of the phenotypic variation (Table 4). Two additional QTL were detected on 2A and 4AL at which the allele of the durum cultivar Karur conferred resistance. These QTL were found in single greenhouse experiments only and, contrary to the QTL at the *Fhb1* locus, were not considered as stable.

**Table 4:** Locations and estimates of QTL for FHB spread (percent of infected spikelets PIS) using multiple QTL mapping

Experiment	Chr.	Closest marker	Add^a^	%PV^b^	LOD^c^
GH1	2A	1698827	−4.9	13.5	4.7
GH1	3BS	Xbarc147	14.6	33.6	10.2
GH2	3BS	1032004	4.9	14.6	3.8
GH3	3BS	Xbarc147	4.7	10.3	3.0
Overall mean	3BS	Xbarc147	7.4	33.3	9.7
GH3	4AL	1235993	−5.2	12.6	3.6

^a^Positive additive effects denote trait-increasing effect of the DBC-480 allele; additive effects were estimated as half the difference between phenotype averages for the homozygote

^b^Percentage of phenotypic variance explained by the QTL

^c^LOD (logarithm of the odds) above the threshold at the 0.05 level of probability obtained through a 1000-iteration permutation test

#### QTL analysis for plant height and flowering date

QTL for plant height were detected on 4AL, 4BS and 6AS to which DBC-480 alleles contributed to increased height (Table 5). The *Rht*-*B1* locus on 4BS was significant in all populations and explained 95, 81 and 37% of the variation for PH in the KD, DD and SD population. In the SD population, two further QTL were associated with PH on 4AL and 6AS. The main effects were 11 and 27% for 4AL and 6AS, while epistatic interaction was evident for both loci with the *Rht*-*B1* locus. The percentage of variation explained by the interaction of 4ALx4BS and 6ASx4BS was low compared to the main effects of each locus contributing to 4 and 7% of the phenotypic variance. QTL detection for flowering date revealed significant QTL on 1BL, 2BS, 4AL, 5AL, 6AS and 6BS (Table 5). The DBC-480 allele contributed to a delayed flowering of RILs carrying it on QTL 2BS, 4AL, 5AL, 6AS and 6BS. The QTL on 2BS was found at the same position in all three populations and mapped at a location distal from the QTL for FHB severity identified in the KD population on 2BL. This QTL had the strongest effect in the DD population where it explained 44% of the phenotypic variation. In the SD population, co-localization of QTL for FHB severity, PH and date of anthesis were found on 4AL and 6AS and thus these loci appeared to have pleiotropic effects.

**Table 5:** Locations and estimates of QTL for plant height (cm) and flowering date (days after May 1st) using multiple QTL mapping

Trait	Population	CHR	Closest marker	Add^a^	%PV^b^	LOD^c^
Plant height	SD	4AL	4541598	−5.6	10.8	22.7
	KD	4BS	RhtB1	−17.4	95.4	74.4
	DD	4BS	RhtB1	−14.9	81.4	36.2
	SD	4BS	RhtB1	−11.0	37.0	45.8
	SD	6AS	4008755	−9.0	27.2	38.6
	SD	4BSx4AL		3.6	3.6	9.5
	SD	4BSx6AS		4.5	7.3	16.3
Flowering date	DD	1BL	4009852	−0.3	6.9	3.6
	KD	2BS	1238155	0.3	11.6	5.0
	DD	2BS	4404789	0.8	44.3	16.8
	SD	2BS	988615	0.4	10.8	3.9
	SD	4AL	4541598	0.5	16.6	5.7
	KD	5AL	1148774	−0.6	37.8	13.6
	SD	6AS	4008755	0.5	20.4	6.8
	DD	6BS	1077913	0.3	7.1	3.7

^a^Positive additive effects denote trait-increasing effect of the DBC-480 allele; additive effects were estimated as half the difference between phenotype averages for the homozygote

^b^Percentage of phenotypic variance explained by the QTL

^c^LOD (logarithm of the odds) above the threshold at the 0.05 level of probability obtained through a 1000-iteration permutation test

### Association between FHB resistance QTL and plant height QTL

**Fig. 3:**
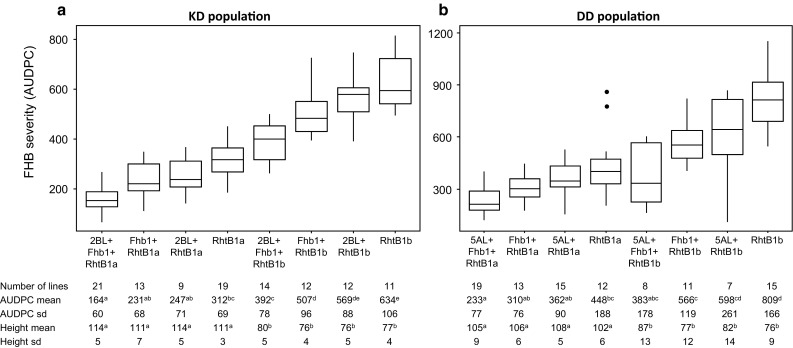
Box plot distributions of RILs according to their allele combinations at the FHB resistance loci for the KD (**a**) and DD (**b**) populations for overall mean FHB severity (AUDPC). Medians are indicated by *solid lines*, *points* represent outliers. For each subgroup, the number of lines, mean values and standard deviations of FHB severity (AUDPC) and plant height (cm) are indicated. Values followed by different letters are significantly different (*p* < 0.05) based on Tukey test

The target genotype for a durum wheat breeder is a semi-dwarf plant type with improved FHB resistance. To investigate the feasibility of such an ideotype in our populations, we compared the effects of the detected FHB resistance QTL. In the SD population the three major resistance QTL mapping to *Rht*-*B1*, 4AL and 6AS overlapped with the QTL for PH. The effect of *Fhb1*—the only QTL not associated with PH in this population—does not efficiently counteract the increased susceptibility associated with the short-stemmed allele at the *Rht*-*B1,* 4AL and 6AS loci. In the KD and DD populations, only the *Rht*-*B1* loci contributed to both FHB severity and PH, while two other QTL, including *Fhb1*, were not associated with this morphological trait. To investigate the effects of allele combinations at the FHB resistance loci on FHB severity and PH, the RILs of each population were first classified in subgroups according to their allele status at the detected resistance QTL as illustrated in Fig. 3. The resistance level and average height were then compared among the different subgroups. In both populations, lines carrying the dwarfing allele *Rht*-*B1b* were significantly shorter and more susceptible than the ones harboring the wild-type allele *Rht*-*B1a*. Plant height on average was reduced by 31 and 25% in the KD and DD *Rht*-*B1b* subpopulations, respectively, but the level of disease symptoms relative to *Rht*-*B1a* were about twofold increased. In the KD population, lines carrying both resistance QTL at *Fhb1* and 2BL loci in combination with *Rht*-*B1b* had between 22 and 38% less disease severity than the dwarf lines carrying one or no resistance QTL while showing equivalent levels of resistance as lines carrying *Rht*-*B1a* with no supplementary resistance QTL. In the DD population, the FHB resistance levels of dwarf lines carrying positive alleles at *Fhb1* and 5AL were not significantly different from any *Rht*-*B1a* subpopulation while being 53% less diseased than dwarf lines with no resistance QTL. Taken together, our findings demonstrate that the combination of 3BS + 2BL QTL in the KD population and 3BS + 5AL in the DD population efficiently offset the negative effect of *Rht*-*B1b* on FHB resistance.

## Discussion

FHB resistance has become a priority in durum wheat breeding programs in the last decades. The limited sources of resistance available in durum wheat have urged breeders in incorporating FHB resistance from related species. In this research work, resistance derived from common wheat was investigated for the first time in the genetic background of durum wheat. The resistant line DBC-480, which carries the major common wheat resistance QTL *Fhb1*, presented enhanced resistance in field and greenhouse experiments after artificial inoculation. The use of three different F7-RIL populations allowed the dissection of the genetic basis of FHB resistance and to concomitantly validate the effects of the detected QTL in the different elite durum backgrounds.

Quantitative variation for FHB symptoms was evident in all three populations and for both inoculation techniques. FHB severity and FHB spread were significantly, but weakly correlated. This low correlation between the two FHB-related traits may be explained by the different mechanisms of infection accounted for by the two inoculation methods. FHB severity assessed after spray inoculation in the field accounts for both resistance to primary infection and subsequent spread of the symptoms within the heads. This measure evaluates thus a combination of type 1 and type 2 resistance under conditions that mimic natural epidemics, while single-floret inoculation estimates solely type 2 component of resistance. Reports have shown that type 1 and type 2 resistance vary independently among cultivars (Schroeder and Christensen 1963) and are likely controlled by different genes (Buerstmayr et al. 2003a). Additionally, a high negative correlation between plant height and FHB resistance was evident in the field trials, while no association between these traits was observed in the greenhouse experiments. The discrepancy of plant height influencing FHB response between the two inoculation methods may also contribute to this low correlation, as several reports have pointed out that type 2 resistance is less affected by plant height than type 1 resistance (Steiner et al. 2004; Srinivasachary et al. 2008, 2009; Lu et al. 2011).

### Genetic architecture of FHB resistance

The genetic architecture of FHB resistance in our populations appears to be quantitative and oligo- to polygenic. A total of six QTL located on chromosome arms 2BL, 3BS, 4AL, 4BS, 5AL and 6AS were repeatedly associated with enhanced resistance and DBC-480 contributed the favorable alleles at all loci. Genotyping of the populations was performed using GBS DArTseq marker technology supplemented with DNA markers specific to *Fhb1* and *Rht*-*B1*. Comparisons of QTL positions were performed based on the consensus wheat map provided by DArT PL (A. Kilian, Diversity Arrays Technologies, personal communication, 2016), which includes DArTseq GBS, DArT and SSR markers, and consensus maps published by Somers et al. (2004) and Marone et al. (2012). It appears that the genomic regions found to be responsible for FHB resistance in our study coincide with locations where QTL have already been identified in common wheat.

The QTL on 3BS mapped at the *Fhb1* locus near *Xbarc147* and *Xumn10* was found repeatedly in all populations. The effect of *Fhb1* on FHB severity varied and, depending on the durum genetic background and the individual experiments, explained between 5 and 16% of the phenotypic variance. In the different populations, the *Fhb1* resistance allele reduced FHB severity symptoms on average by 30% in the KD and 36% in the DD populations, while in the SD population the resistance was only increased by 6%. The discrepancies observed among the KD and DD populations on one side, and the SD population on the other, may be due to differences in their respective resistance genetic architecture. In the KD and DD populations, only one further major QTL affecting FHB severity was detected and similar effects for *Fhb1* were observed, while in the SD population the relative effect of *Fhb1* may be diminished by the presence of three further major QTL. When evaluating FHB spread after single-floret inoculation, the *Fhb1* locus had a large effect explaining 33% of the total phenotypic variance. Our study demonstrates that in durum wheat, *Fhb1* is effective in providing type 2 resistance in a similar way as established in common wheat where *Fhb1* improves mainly type 2 resistance and to a lesser extent type 1 resistance (Waldron et al. 1999; Anderson et al. 2001; Buerstmayr et al. 2002, 2003a; Cuthbert et al. 2006). *Fhb1* is a well-characterized QTL descending from the Asian cultivar Sumai-3 which has been found in numerous QTL studies (Buerstmayr et al. 2009). In tetraploid wheat, a resistance QTL has also been found in proximity of the *Fhb1* genomic region in the durum cultivar Floradur (Buerstmayr et al. 2012) and in *Tunisian durum* landraces (Ghavami et al. 2011). However, haplotype comparison using SSR markers by Buerstmayr et al. (2012) revealed different alleles for Sumai-3 and Floradur at the *Fhb1* locus, indicating thus the existence of different QTL alleles at this locus. We report here the first successful deployment of *Fhb1* in durum wheat which marks a significant step forward in durum wheat breeding toward improving FHB resistance. Common wheat represents a useful reservoir of resistance for durum wheat, as most of the QTL that have been identified are located on the A and B genomes (Buerstmayr et al. 2009; Liu et al. 2009; Löffler et al. 2009). The difficulties pointed out in previous studies when transferring resistance QTL from common wheat into durum wheat may be attributed to complex interactions of genes among the A, B and D genomes as emphasized in a recent study by Zhu et al. (2016a). In our study, no epistatic interactions that may modulate the effect of *Fhb1* were detected. The absence of the D genome in durum wheat, hypothesized to carry factors that enhance resistance (Fakhfakh et al. 2011), has been speculated as one of the limiting factors for effective deployment of resistance from hexaploid wheat. Our results show that the presence of the D genome appears to be not required for efficient expression of *Fhb1* in durum wheat. Comparing the effect of *Fhb1* in durum wheat with previous studies is not trivial, due to a wide range of phenotypic variances reported for this QTL in common wheat (Buerstmayr et al. 2009). In studies evaluating type 2 resistance in common wheat, *R* ^2^ values for *Fhb1* ranged between 11% (Yang et al. 2005) and 60% (Bai et al. 1999), while in spray-inoculated field trials Buerstmayr et al. (2003a) reported an *R* ^2^ of 29%. The resistance-improving effect of *Fhb1* in our durum wheat populations is in a similar range as reported in a series of near isogenic lines in common wheat by Pumphrey et al. (2007), who found an average reduction of disease severity by 23%, though varying from 0 to 70%. Differences in population size, genetic background, inoculation techniques and environments in which the evaluations were performed can all be reasons for these variations. While in the literature, there are speculations that durum wheat may carry or lack certain genetic factors that modulate the resistance-improving effect of *Fhb1* (Rudd et al. 2001), we find no evidence to support this hypothesis.

A major QTL on 4BS associated with FHB severity was found in all three populations with effects of different magnitude. The location of the QTL coincided with the *Rht*-*B1* gene. The QTL was responsible for the greatest amount of variation for resistance in the KD and DD populations, while in the SD population the QTL had a major effect but was not the greatest contributor to FHB resistance. As mentioned previously, the discrepancy of effects observed is certainly due to differences in genetic backgrounds. In all cases, the reduced height allele *Rht*-*B1b* accounted for higher disease severity. Such associations of the semi-dwarf *Rht*-*B1b* allele with increased FHB severity have been previously reported in hexaploid wheat (Hilton et al. 1999; Srinivasachary et al. 2009). Supporting results have also been found in three durum wheat backcross populations from crosses of the tall and FHB-resistant donor *T. dicoccum*-line 161 to the semi-dwarf durum wheat lines Helidur, Floradur and DS-131621. The resistance QTL at the *Rht*-*B1* locus was the most important QTL affecting FHB resistance after spray inoculation and in all three populations, plants carrying the *Rht*-*B1b* allele showed higher FHB severity scores (Buerstmayr et al. 2012).

In the SD population, two further major QTL were detected on 4AL and 6AS. Both resistance QTL overlapped with QTL for flowering date and plant height. QTL have been already identified on 4A and 6A in tetraploid wheat; however, they appear to be located on different chromosome arms and do not match the position of the QTL identified in our study. Gladysz et al. (2007) identified a QTL for type 2 resistance derived from the resistant *T. dicoccoides* accession Mt.Hermon#22 near *Xgwm610* which mapped on the short arm of chromosome 4A, while Buerstmayr et al. (2012) found a small effect QTL for FHB severity derived from *T.dicoccum*-line 161 in a cross with line DS-131621 near *Xgwm356* on 6AL. Meanwhile, several mapping projects performed in hexaploid wheat identified QTL in the same region of 4AL in the US winter wheat Heyne (Zhang et al. 2012) and in the Swiss winter wheat Arina (Paillard et al. 2004; Buerstmayr and Buerstmayr 2015). No coinciding QTL for flowering date or plant height were reported, but an overlap with QTL for anther retention was found in the Arina/Capo population (Buerstmayr and Buerstmayr, 2015). The QTL on 6AS mapped to a similar position as the type 2 resistance QTL identified in hexaploid wheat in the ND2603/Butte86 population derived from the resistant line ND2603 (Sumai 3/Wheaton) (Anderson et al. 2001).

In the KD population, a minor effect QTL was found on 2BL at the proximity of the centromere. This region has been reported to carry resistance QTL in two unrelated tetraploid wheat populations where the susceptible durum wheat parents Strong field (Somers et al. 2006) and Helidur (Gladysz et al. 2007) contributed to the resistance-improving allele. These QTL were detected after point inoculation providing type 2 resistance, while the QTL identified in our study was found after spray inoculation.

Another QTL for FHB severity was identified on 5AL in the DD population, which had a major effect in 2013 but remained undetected in 2014 and 2015. In the DD population, very few GBS markers were polymorphic on chromosome 5A making exact positioning of the QTL difficult. To improve map density, 21 SSR markers were additionally screened, yet none was found to be polymorphic suggesting close genetic relatedness of the parental lines for this genomic region. Comparison with previous studies is therefore difficult, but map comparison suggests that the QTL does not map to the same region as the major hexaploid wheat QTL on 5A *Qfhs.ifa*-*5A* derived from Sumai-3 which is located close to the centromere (Buerstmayr et al. 2003a).

### Association of QTL for FHB resistance, flowering date and plant height

In our study six QTL were found associated with flowering date. Co-localization of QTL for flowering date and FHB resistance was evident for the SD population on 4AL and 6AS. The two QTL exert a strong effect on both traits for which a positive correlation was observed. In the KD and DD populations, weak and non-significant correlations were found, and when individual experiments were analyzed separately, the correlations varied greatly. No general pattern was evident for the association between earliness and the level of FHB symptoms in these two populations. This non-dependency may be attributed to the absence of overlapping QTL for these two traits, while environment-specific factors around flowering and inoculation may account for variability in the correlations observed in individual experiments.

In contrast, plant height was significantly negatively correlated with FHB severity in all three populations, which is in agreement with previous findings (Talas et al. 2011; Buerstmayr et al. 2012; Miedaner and Longin 2014). All PH QTL identified in this study coincided with QTL for FHB severity on chromosomes 4AL, 4BS and 6AS. Co-localization of PH and FHB severity QTL is a common feature in wheat and supported by meta-QTL analysis (Mao et al. 2010). The mechanisms of association between the two traits are complex and may be attributed to the effects of height differences per se and/or to pleiotropic effects of the dwarfing genes or tightly linked genes that increase FHB susceptibility. The mutant allele *Rht*-*B1b,* as well as its homoeologous allele *Rht*-*D1b* on chromosome 4D, encodes single nucleotide polymorphism (SNP) mutations in the DELLA domain that create a premature stop codon that is responsible for reduced sensitivity to the phytohormone gibberellin leading to shorter plant height (Peng et al. 1999; Hedden and Sponsel 2015). DELLA proteins have been shown to be associated with abiotic and biotic stress tolerance (Achard and Genschik 2009) and, in the case of FHB, a DELLA protein mutation may have physiological effects linked to changes in cell death response (Saville et al. 2012). Alternatively to these genetic effects, differences in microclimatic conditions around the heads of tall and dwarf genotypes have been considered to play a significant role particularly under field conditions, with short plants being exposed to higher infection pressure than tall plants (Yan et al. 2011). The *Rht*-*B1b* allele is also known to have pleiotropic effects on different morphological and structural traits including reduced peduncle length and increased cell density, which may also affect response to FHB. In common wheat, *Rht*-*B1b* and *Rht*-*D1b* were found to be associated with reduced anther extrusion, which was supposed to partly explain their association with higher FHB susceptibility (Buerstmayr and Buerstmayr 2016). In the case of our study, all features may be important. The strong effect of the QTL on 4BS, coinciding with the reduced height gene *Rht*-*B1,* on PH and FHB severity was evident in all populations. The *Rht*-*B1* locus explained 95, 81 and 37% of the variation for PH and 64, 38 and 19% of the variation for FHB severity in the KD, DD and SD populations, respectively. In the SD population, the two additional QTL on 4AL and 6AS exerted at the same time strong effect on plant height and FHB resistance. The QTL on 4AL mapped to a similar region as a QTL associated with PH in hexaploid wheat corresponding to a kaurenoic acid oxidase (KAO) gene (Khlestkina et al. 2010; Zanke et al. 2014), while reduced height genes have been reported on 6AS in durum wheat (Haque et al. 2011). These genes are gibberellin-sensitive and not comparable to *Rht*-*B1* in that aspect. In agreement with Yan et al. (2011), we hypothesize that considering the large variation for plant height with differences of about 60 cm between the shortest and the tallest plants, part of the apparent negative correlation, may be attributed to plant height per se. Even under spray inoculation and mist irrigation, heads of short plants tend to remain more humid and therefore under more severe infection pressure than heads of tall plants. This is in agreement with Buerstmayr et al. (2012), who evaluated FHB severity of three durum wheat populations with similarly large variation for plant height as observed in our present study and argued for a probable disease escape of tall lines despite controlled mist irrigation after spray inoculation.

### Perspective for durum wheat breeding and conclusion

Obviously, in our populations, plant height had a strong influence on modulating FHB disease response. The increased FHB susceptibility associated with medium to short height plant and with *Rht*-*B1b* is challenging for durum wheat breeders. *Rht*-*B1b* confers beneficial attributes linked to higher yield and harvest index concomitant to the desired reduced plant height, thereby limiting lodging, in plant production systems with modern agronomic practices (Royo et al. 2007; Subira et al. 2016). We show that the successful deployment of *Fhb1* in combination with minor effect QTL enabled the discovery and the selection of semi-dwarf lines with upsurge levels of resistance. These results are in agreement with a previous report in hexaploid wheat where pyramiding two resistance QTL balanced the negative effect of the semi-dwarf allele *Rht*-*D1b* to achieve improved levels of resistance in semi-dwarf wheat (Lu et al. 2011). The progeny lines of the KD and DD populations carrying favorable allele combinations at the *Rht*-*B1* and *Fhb1* loci, and additional FHB resistance alleles on 2BL or 5AL provide unique and novel resources for durum wheat breeding. The introgression of *Fhb1* by recurrent backcrossing into durum wheat to develop the resistant experimental line DBC-480 and its crossing to elite durum cultivars enabled the development of novel FHB-resistant breeding lines that are agronomically close to modern European germplasm. These novel improved lines are thus readily incorporable into practical durum wheat breeding programs for enhancing FHB resistance.

#### Author contribution statement

NP performed the study and drafted the manuscript. CG, UP and EW helped to collect phenotypic data and genotyping of markers. BS carried out the initial crosses and developed the plant material used in this study. TL, OR and HB initiated the study and obtained funding. The manuscript was finally approved by all coauthors.

## Electronic supplementary material

Below is the link to the electronic supplementary material.

Supplementary material 1 (PDF 37 kb)

Supplementary material 2 (PDF 36 kb)

Supplementary material 3 (PDF 201 kb)

Supplementary material 4 (XLSX 395 kb)

Supplementary material 5 (PDF 123 kb)
