# Supplementary material for: QTL mapping of Fusarium head blight resistance in three related durum wheat populations
Source: Theor Appl Genet. 2016 Sep 23;130(1):13–27. doi: 10.1007/s00122-016-2785-0 (PMC5215227; doi:10.1007/s00122-016-2785-0)
Supplement: Supplementary file 1 — Supplementary material 1 (PDF 37 kb) [file 122_2016_2785_MOESM1_ESM.pdf]

Article title: QTL mapping of Fusarium head blight resistance in three related durum wheat populations

Journal: Theoretical and Applied Genetics

Authors: Prat Noemie[1,2,3], Guilbert Camille[1], Prah Ursa[1], Wachter Elisabeth[1], Steiner Barbara[1], Langin Thierry[2], Robert Olivier[3], Buerstmayr Hermann[1]

[1] University of Natural Resources and Life Sciences Vienna, Department of Agrobiotechnology, Institute of Biotechnology in Plant Production, Konrad Lorenz Str. 20, A-3430 Tulln, Austria
[2] GDEC, INRA, UBP, 63039, Clermont-Ferrand cedex 2, France
[3] Florimond-Desprez, 3 rue Florimond-Desprez, BP 41, 59242 Cappelle-en-Pevele, France

Author for correspondence: hermann.buerstmayr@boku.ac.at

ESM1_A Variance component estimates of genotype $\sigma^2_{Genotype}$, year σ^2_{Year}, block within year $\sigma^2_{Block\ within\ Year}$, genotype × year $\sigma^2_{Genotype×Year}$ and the residual effects σ^2_{error} for FHB severity (AUDPC), plant height (cm) and flowering date (days after May 1st) accross three experiments for the KD, DD and SD populations

Karur x DBC-480 (KD) population

Trait	Variance component				
	$\sigma^2_{Genotype}$	σ^2_{Year}	$\sigma^2_{Block\ within\ Year}$	$\sigma^2_{Genotype×Year}$	σ^2_{error}
FHB severity	28156.91 ***	17626.10	1939.89	5176.38	13615.78
Plant height	321.42 ***	139.98	2.71	12.33	25.13
Flowering date	0.58 ***	55.52	1.47	0.45	1.11

*** p< 0.001

Durobonus x DBC-480 (DD) population

Trait	Variance component				
	$\sigma^2_{Genotype}$	σ^2_{Year}	$\sigma^2_{Block\ within\ Year}$	$\sigma^2_{Genotype×Year}$	σ^2_{error}
FHB severity	43582.79 ***	88140.07	8915.16	28239.04	22843.86
Plant height	252.07 ***	109.17	3.35	12.57	31.58
Flowering date	0.92 ***	52.45	3.24	0.41	1.27

*** p< 0.001

SZD1029K x DBC-480 (SD) population

Trait	Variance component				
	$\sigma^2_{Genotype}$	σ^2_{Year}	$\sigma^2_{Block\ within\ Year}$	$\sigma^2_{Genotype×Year}$	σ^2_{error}
FHB severity	52400.75 ***	261620.52	7280.05	46452.49	20393.17
Plant height	386.28 ***	84.10	8.06	6.83	23.87
Flowering date	1.06 ***	42.72	1.85	0.33	1.11

*** p< 0.001

ESM1_B Variance component estimates of genotype $\sigma^2_{Genotype}$, Experiment σ^2_{Exp}, and the residual effects σ^2_{error} for FHB spread (percent infected spikelets PIS) and plant height (cm) in the KD population accross three unreplicated experiments

Karur x DBC-480 (KD) population

Trait	Variance component		
	$\sigma^2_{Genotype}$	σ^2_{Exp}	σ^2_{error}
FHB spread	84.10 ***	43.17	247.26
Plant height	609.11 ***	15.66	55.74

*** p< 0.001
