# Supplementary material for: QTL mapping of Fusarium head blight resistance in three related durum wheat populations
Source: Theor Appl Genet. 2016 Sep 23;130(1):13–27. doi: 10.1007/s00122-016-2785-0 (PMC5215227; doi:10.1007/s00122-016-2785-0)
Supplement: Supplementary file 2 — Supplementary material 2 (PDF 36 kb) [file 122_2016_2785_MOESM2_ESM.pdf]

Article title: QTL mapping of Fusarium head blight resistance in three related durum wheat populations

Journal: Theoretical and Applied Genetics

Authors: Prat Noemie[1,2,3], Guilbert Camille[1], Prah Ursa[1], Wachter Elisabeth[1], Steiner Barbara[1], Langin Thierry[2], Robert Olivier[3], Buerstmayr Hermann[1]

[1] University of Natural Resources and Life Sciences Vienna, Department of Agrobiotechnology, Institute of Biotechnology in Plant Production, Konrad Lorenz Str. 20, A-3430 Tulln, Austria
[2] GDEC, INRA, UBP, 63039, Clermont-Ferrand cedex 2, France
[3] Florimond-Desprez, 3 rue Florimond-Desprez, BP 41, 59242 Cappelle-en-Pevele, France

Author for correspondence: hermann.buerstmayr@boku.ac.at

ESM2 Pearson correlation coefficients between FHB severity, FHB spread, plant height and flowering date in individual experiments for the KD, DD and SD populations

Karur x DBC-480 (KD) population

	FHB severity (AUDPC)		
	2013	2014	2015
Plant height	-0.65***	-0.71***	-0.85***
Flowering date	0.16 ns	-0.27 ***	-0.05 ns

** p< 0.01
*** p< 0.001
n.s non significant

Durobonus x DBC-480 (DD) population

	FHB severity (AUDPC)		
	2013	2014	2015
Plant height	-0.55***	-0.51***	-0.65***
Flowering date	0.04 ns	-0.19 ns	0.43 ***

** p< 0.01
*** p< 0.001
n.s non significant

SZD1029K x DBC-480 (SD) population

	FHB severity (AUDPC)		
	2013	2014	2015
Plant height	-0.63***	-0.7***	-0.86***
Flowering date	0.33**	0.11 ns	0.66***

** p< 0.01
*** p< 0.001
n.s non significant
