# Supplementary material for: QTL mapping of Fusarium head blight resistance in three related durum wheat populations
Source: Theor Appl Genet. 2016 Sep 23;130(1):13–27. doi: 10.1007/s00122-016-2785-0 (PMC5215227; doi:10.1007/s00122-016-2785-0)
Supplement: Supplementary file 3 — Supplementary material 3 (PDF 201 kb) [file 122_2016_2785_MOESM3_ESM.pdf]

Article title: QTL mapping of Fusarium head blight resistance in three related durum wheat populations

Journal: Theoretical and Applied Genetics

Authors: Prat Noemie[1,2,3], Guilbert Camille[1], Prah Ursa[1], Wachter Elisabeth[1], Steiner Barbara[1], Langin Thierry[2], Robert Olivier[3], Buerstmayr Hermann[1]

[1] University of Natural Resources and Life Sciences Vienna, Department of Agrobiotechnology, Institute of Biotechnology in Plant Production, Konrad Lorenz Str. 20, A-3430 Tulln, Austria
[2] GDEC, INRA, UBP, 63039, Clermont-Ferrand cedex 2, France
[3] Florimond-Desprez, 3 rue Florimond-Desprez, BP 41, 59242 Cappelle-en-Pevele, France

Author for correspondence: hermann.buerstmayr@boku.ac.at

ESM3 Marker distribution in the KD, DD and SD populations

Karur x DBC-480 (KD) population

Chr.	LG[a]	Number of markers	UP[b]	av. dist. (cM)[c]	Total cM mapped
1A	2	318	93	1.8	187.6
1B	2	490	117	1.6	188.9
2A	3	897	135	1.3	207.4
2B	3	692	160	1.3	202.8
3A	3	307	75	2.3	157.6
3B	4	856	127	1.6	185.4
4A	2	686	140	1.8	219.8
4B	1	589	154	1.4	207.7
5A	2	218	100	2.4	221.3
5B	3	412	118	1.9	225.5
6A	1	705	84	2.7	221.5
6B	1	711	96	1.8	174.9
7A	2	318	61	2.3	169.7
7B	2	776	149	3	236.5
Total	31	7975	1609	1.9	2806.6

Durobonus x DBC-480 (DD) population

Chr.	LG[a]	Number of markers	UP[b]	av. dist. (cM)[c]	Total cM mapped
1A	3	91	35	1.1	47.3
1B	2	431	121	1.6	227.1
2A	2	309	68	1.6	108.7
2B	3	935	233	1.4	340.3
3A	1	91	13	1.1	12.6
3B	2	200	91	2	163.6
4A	1	61	27	2.8	72
4B	1	201	49	1.6	77.2
5A	3	65	21	1.6	32.1
5B	2	141	41	2	75.5
6A	1	626	133	2	266.4
6B	1	541	137	1.8	244.9
7A	2	24	15	2.4	36.3
7B	2	437	68	1.5	77.8
Total	26	4153	1052	1.7	1781.8

SZD1029K x DBC-480 (SD) population

Chr.	LG[a]	Number of markers	UP[b]	av. dist. (cM)[c]	Total cM mapped
1A	2	129	20	2.8	41.5
1B	1	292	42	2.7	111.5
2A	2	619	69	1.9	125.5
2B	2	609	43	3.1	125.4
3A	2	60	22	2.9	55.4
3B	3	623	113	1.8	207.5
4A	3	728	125	2.1	226.5
4B	1	351	93	1.9	178.7
5A	2	148	53	3.1	136.4
5B	3	412	77	2.5	189.5
6A	2	492	65	2.3	166
6B	1	714	116	1.9	216.8
7A	2	322	50	4	191.1
7B	2	743	118	2.2	252.5
Total	28	6242	1006	2.5	2224.3

[a] Number of linkage groups in each chromosome
[b] Number of unique positions
[c] average distance between adjacent markers
