# Supplementary material for: QTL mapping of Fusarium head blight resistance in three related durum wheat populations
Source: Theor Appl Genet. 2016 Sep 23;130(1):13–27. doi: 10.1007/s00122-016-2785-0 (PMC5215227; doi:10.1007/s00122-016-2785-0)

Article title: QTL mapping of Fusarium head blight resistance in three related durum wheat populations

Journal: Theoretical and Applied Genetics

Authors: Prat Noemie[1,2,3], Guilbert Camille[1], Prah Ursa[1], Wachter Elisabeth[1], Steiner Barbara[1], Langin Thierry[2], Robert Olivier[3], Buerstmayr Hermann[1]

[1]Department for Agrobiotechnology Tulln, BOKU-University of Natural Resources and Life Sciences, ienna, Konrad Lorenz Str. 20, 3430 Tulln, Austria

[2]GDEC, INRA, UBP, 63039, Clermont-Ferrand cedex 2, France

[3]Florimond-Desprez, 3 rue Florimond-Desprez, BP 41, 59242 Cappelle-en-Pevele, France

Corresponding author: hermann.buerstmayr@boku.ac.at

ESM5_A Boxplot distributions of RILs according to their allele status at *Fhb1* (A) and *Rht-B1* (B) loci for overall mean FHB severity (AUDPC) for each population. Medians are indicated by solid lines. For each group, the number of lines is indicated

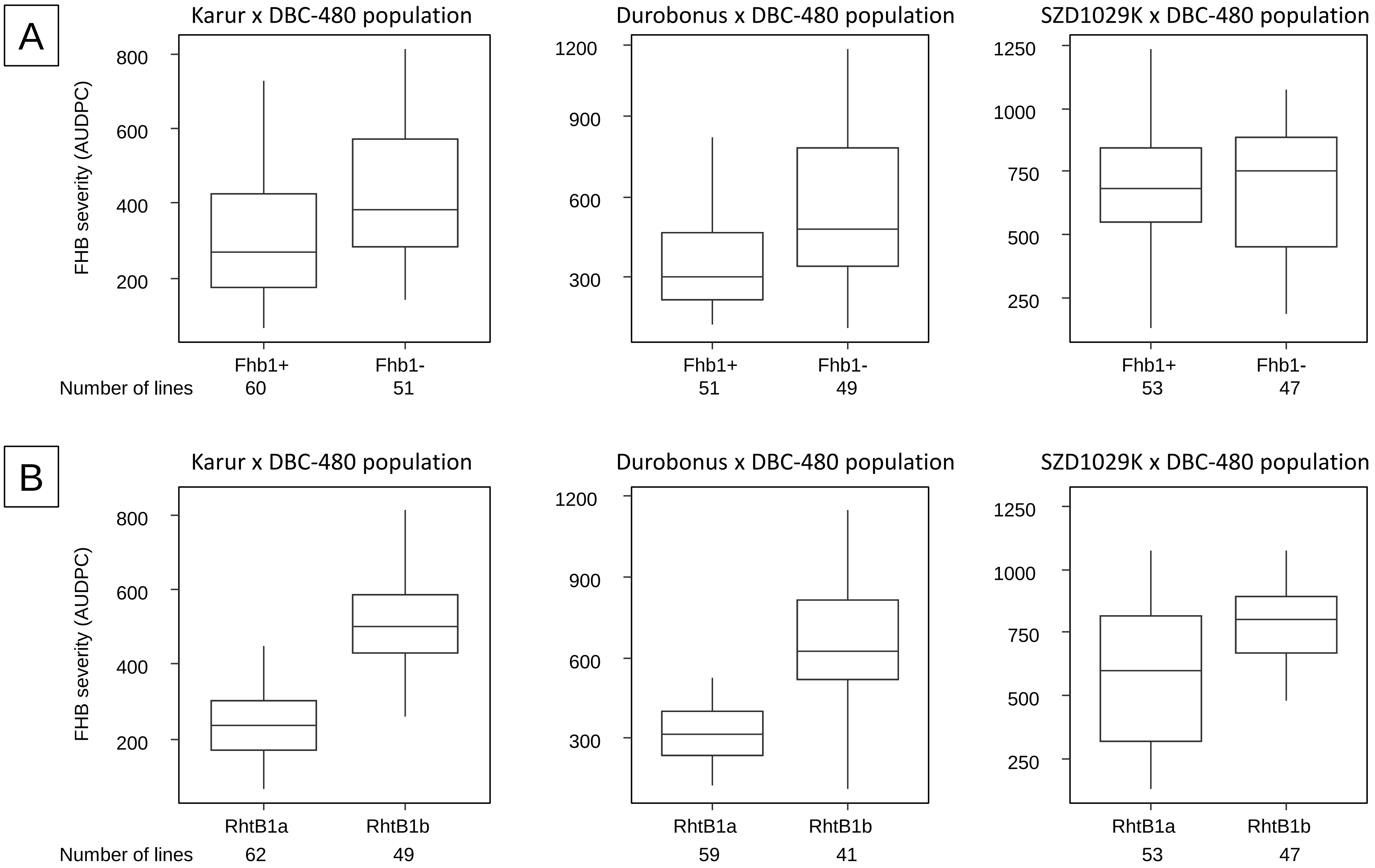

ESM5_B Boxplot distributions of RILs according to their allele combinations at *Fhb1* and *Rht-B1* loci for overall mean FHB severity (AUDPC) for each population. Medians are indicated by solid lines. For each subgroup, the number of lines is indicated

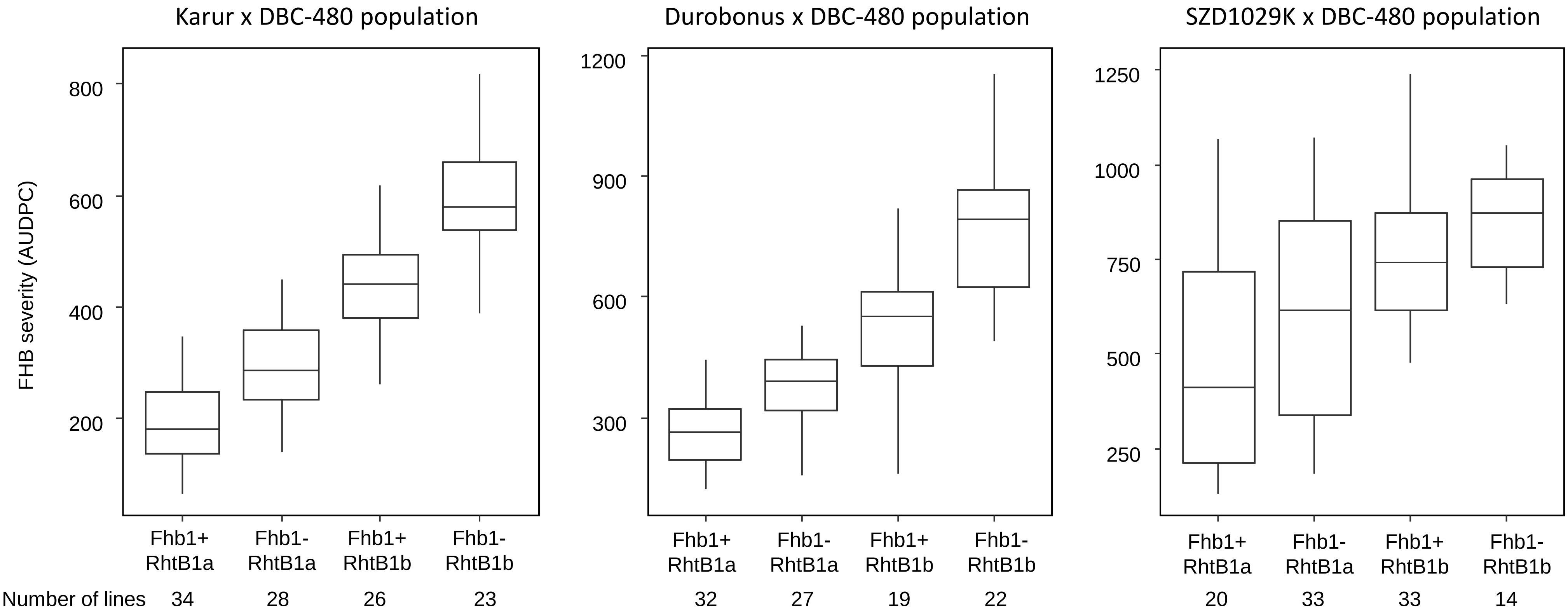

Supplement: Supplementary file 5 — Supplementary material 5 (PDF 123 kb) [file 122_2016_2785_MOESM5_ESM.pdf]
